# Dysmorphological and Neuropsychological Phenotypes of Prenatally Alcohol‐Exposed 6‐Year‐Old Children: A Prospective Longitudinal Birth Cohort Study

**DOI:** 10.1111/acer.70320

**Published:** 2026-05-13

**Authors:** M. Jolma, E. Wallén, E. Saure, K. Rämö, H. Kahila, N. Kaminen‐Ahola

**Affiliations:** ^1^ Division of Child Neurology Päijät‐Häme Central Hospital Lahti Finland; ^2^ Faculty of Medicine University of Helsinki Helsinki Finland; ^3^ Environmental Epigenetics Laboratory, Department of Medical and Clinical Genetics, Medicum, Faculty of Medicine University of Helsinki Helsinki Finland; ^4^ BABA Center, Pediatric Research Center Helsinki University Hospital and University of Helsinki Helsinki Finland; ^5^ Department of Obstetrics and Gynecology Helsinki University Hospital and University of Helsinki Helsinki Finland

**Keywords:** balance, cognition, dysmorphism, early exposure, fetal alcohol spectrum disorder, prenatal alcohol exposure

## Abstract

**Background:**

Prenatal alcohol exposure (PAE) disrupts embryonic development, resulting in variable fetal alcohol spectrum disorder (FASD) phenotypes characterized by dysmorphological and neurodevelopmental abnormalities. To elucidate the etiology of FASD, we established a prenatally recruited prospective birth cohort collecting biological samples at birth and longitudinal data from alcohol‐exposed and unexposed control children.

**Methods:**

Dysmorphological, neuropediatric, and neuropsychological assessments were performed at 6 years of age in 28 children with substantial PAE and 52 control children.

**Results:**

Among the PAE group, 76% (19/25) of the children who completed the full evaluation met the diagnostic criteria for FASD, including five with fetal alcohol syndrome (FAS), six with partial FAS, seven with alcohol‐related neurodevelopmental disorder, and one with alcohol‐related birth defect. None of the tested children had a prior FASD diagnosis. In addition to the characteristic features of FAS, including short palpebral fissures, smooth philtrum, and thin vermilion border, several additional anomalies, such as mild midface hypoplasia, were significantly associated with PAE (*p* < 0.001). Also, PAE was associated with both underweight and overweight at 6 years of age, particularly among boys. Neurocognitive assessments revealed lower performance scores and a high prevalence of ADHD symptoms in PAE children compared with controls. Furthermore, PAE children exhibited additional phenotypic features not captured by the diagnostic FASD criteria, including impairments in adaptive and social functioning, somatic health problems, and reduced postural stability. Moreover, 11 children with early‐only PAE limited to the first seven gestational weeks showed dysmorphic features and cognitive outcomes comparable to those of children with longer exposure.

**Conclusions:**

The high proportion of affected children demonstrates the need for systematic follow‐up of those with PAE and comprehensive phenotypic characterization to improve FASD identification. Importantly, these findings indicate that substantial PAE limited to very early pregnancy is sufficient to result in FASD with clinically significant neurocognitive impairment.

## Graphical abstract

A prospective cohort study assessed 28 children with substantial prenatal alcohol exposure (PAE) and 52 controls at the age of 6. Most PAE children met Fetal Alcohol Spectrum Disorder (FASD) criteria, showing dysmorphic features, neurocognitive deficits, ADHD symptoms, and other health impairments. None had a prior diagnosis, demonstrating the need for systematic follow‐up and comprehensive phenotypic characterization. Notably, PAE limited to the first 7 weeks of pregnancy produced comparable FASD outcomes.

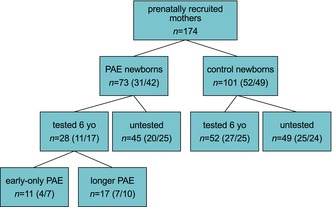

## 1 Introduction

Prenatal alcohol exposure (PAE) can result in a spectrum of lifelong physical, psychiatric, and cognitive impairments collectively referred to as fetal alcohol spectrum disorders (FASD). These disorders are estimated to affect 2%–5% of individuals in the Western countries (Lange et al. 2017; Popova et al. 2017, 2023; Roozen et al. 2016). Individuals with FASD may exhibit a wide range of neurodevelopmental and somatic disorders, including but not limited to attention deficit hyperactivity (ADHD), autism spectrum disorder (ASD), schizophrenia, seizures, visual impairments, and middle‐ear infections (Attell et al. 2025; Popova et al. 2016).

The effects of PAE and the resulting phenotypes in affected offspring depend not only on the amount and frequency of exposure but also on maternal characteristics, such as weight, age, and concurrent smoking, as well as on genetic susceptibility in both the mother and the fetus (Popova et al. 2023). The timing of exposure is also critical, and substantial effects of early exposure during the first weeks of embryonic development have been reported in experimental human cell models and animal studies (Lipinski et al. 2012; Wallén et al. 2021, 2025). However, human studies on the effects of early‐only exposure are still limited. Due to the variable exposures and other risk factors leading to diverse and age‐dependent phenotype, recognition and diagnosis of FASD remain challenging.

Certain dysmorphological, neurodevelopmental, and physiological features associated with PAE are included in the widely used Institute of Medicine (IOM) 2016 diagnostic criteria for FASD (Del Campo et al. 2024; Hoyme et al. 2016). FASD can be classified into four categories according to the IOM criteria: fetal alcohol syndrome (FAS), partial fetal alcohol syndrome (PFAS), alcohol‐related neurodevelopmental disorder (ARND), and alcohol‐related birth defect (ARBD). FAS is considered the most severe form of FASD, characterized by growth restriction, functional or structural brain abnormalities, and typical facial features such as short palpebral fissures, smooth philtrum, and a thin upper lip (Hoyme et al. 2016). The developing nervous system is particularly sensitive to the effects of alcohol (Guerri 2002; Wallén et al. 2025). Indeed, the most common category of FASD appears to be ARND, which manifests primarily as a neurological developmental disorder without growth retardation or the facial features of FAS (Wozniak et al. 2019).

To investigate FASD etiology and the effects of PAE on phenotype, we established the prospective epiFASD cohort through prenatal recruitment, including 73 alcohol‐exposed and 101 control newborns. The cohort includes biological samples, detailed pregnancy and birth information collected at delivery, and longitudinal follow‐up data of the children. To better understand the characteristic patterns of effects associated with PAE and their developmental manifestations, we conducted dysmorphological, neuropediatric, and neuropsychological assessments in 28 children with PAE and 52 unexposed controls, analyzing differences in their phenotypes at 6 years of age related to prenatal exposure information and birth data. This age represents an important developmental stage, as key decisions regarding schooling and the need for supportive interventions are typically made at this time. Furthermore, to evaluate the representativeness of the tested children relative to the full epiFASD cohort, we analyzed information collected from 45 PAE and 49 unexposed control newborns and their mothers who were part of the study cohort but did not participate in the 6‐year assessments.

## 2 Materials and Methods

### 2.1 The epiFASD Cohort

This study investigates the phenotypes of children in the epiFASD cohort, which comprises biological samples and follow‐up information on newborns with PAE as well as unexposed controls. Informed consent was obtained from all participants, and the study received ethical approval (HUS/683/2020) and permission from Helsinki University Hospital (HUS/706/2025). The first 174 children of the cohort born between 2013 and 2018 reached 6 years of age between 2019 and 2024, during which the phenotypic assessments were conducted.

Women with substantial alcohol consumption (*n* = 73) were recruited in a specialized outpatient clinic for pregnant women with substance use problems at Helsinki University Hospital, Finland. Maternal alcohol consumption was assessed using self‐reported measures: the alcohol use disorders identification test (AUDIT) and/or the number of alcohol units consumed per week (ad) (1 unit = 12 g of ethyl alcohol). The AUDIT is a 10‐item screening tool developed by the World Health Organization to estimate alcohol consumption, drinking behavior, and alcohol‐related problems (Babor et al. 2001). Maternal alcohol consumption according to AUDIT scores or ad is presented in three categories describing alcohol use–related risks for nonpregnant women (Rehm et al. 2015; Tynjälä et al. 2012):

Category 1. AUDIT 1–5 or < 7 ad: low risk for nonpregnant women.

Category 2. AUDIT 6–13 or 7–11 ad: harmful/moderate risk for nonpregnant women.

Category 3. AUDIT 14–40 or ≥ 12 ad: high‐risk/likely alcohol dependence.

The timing of alcohol exposure during pregnancy was also self‐reported and classified into three categories: gestational weeks (GW) 1–12, GW 1–28, and GW 1–42 to avoid reporting individual‐level data. For subgroup analyses, children whose mothers consumed alcohol only up to GW 7 were classified as early‐only PAE, and the rest of the children as longer PAE exposure, consistent with our previous study (Auvinen et al. 2022). GW 7 was selected as the cutoff for early exposure, as it represents the average time at which pregnant individuals in Finland first contact prenatal care and are advised to abstain from alcohol.

Control mothers (*n* = 101) were healthy Finnish, Caucasian women recruited from Helsinki University Hospital between 2014 and 2016 by a nurse during antenatal visits or by a physician prior to delivery in hospital. According to their self‐reported information, they did not use alcohol or tobacco during pregnancy. Control children were conceived naturally and delivered spontaneously.

Parents were asked about their willingness to participate in the assessment conducted at the age of six already during the recruitment phase, and letters of invitation were sent to those who responded positively. Most participants were willing to take part in further studies; however, not all control children could be assessed due to limited resources, and some parents of exposed children could not be reached. In addition, several parents of exposed children who were already receiving support declined participation or were ineligible, as their children had recently undergone neuropsychological assessment.

**FIGURE 1:**
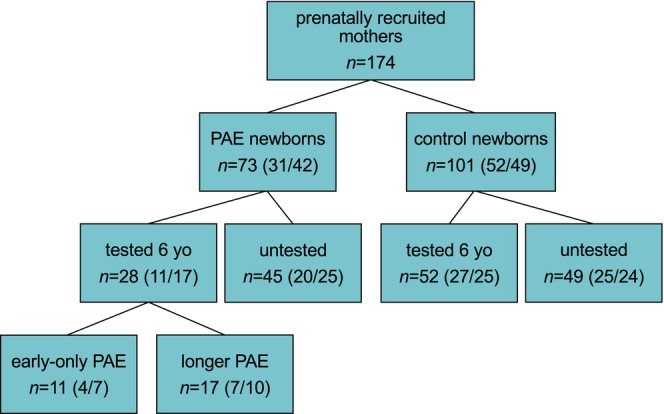
Flowchart of study participants. Numbers of recruited mothers, newborns (PAE vs. control), children tested or not tested at 6 years (6 yo), and those with early‐only PAE versus longer PAE exposure are shown. Sex of the children is indicated in brackets (males/females).

A total of 80 six‐year‐old children from the epiFASD cohort born between 2013 and 2018 participated in dysmorphological, neuropediatric, and neuropsychological assessments between 2020 and 2024: 28 alcohol‐exposed (11 males, 17 females) and 52 controls (27 males, 25 females) (Figure 1). Although not all 28 PAE children completed every assessment, each test included data from at least 25 children. The exposure details were blinded to the pediatric neurologist and neuropsychologist during the evaluations. However, it was evident from the medical history of the child if they had been in foster care, adopted, or followed in the social pediatric unit due to prenatal substance exposure.

Maternal and birth information collected included maternal age, prepregnancy body mass index (BMI), pregnancy weight gain, placental weight, gestational age (GA) at birth, and birth measures: weight, length, and head circumference (HC). Finnish growth charts (Sankilampi et al. 2013) were used to calculate gestational age‐ and sex‐adjusted *Z* scores (SD values). Childhood BMI was converted to International Standardized BMI (ISO‐BMI) to categorize underweight, healthy weight, overweight, and obesity according to adult‐equivalent thresholds (Cole et al. 2000, 2007).

The comparison of general characteristics of the epiFASD cohort including 73 PAE and 101 control newborns and their mothers is presented in Table 1.

**TABLE 1:** General characteristics of the prenatally alcohol‐exposed (PAE) and control children.

		Control children (*n* = 101) mean (±SD)	PAE children (*n* = 73) mean (±SD)	*p* or OR (95% CI, *p*)
**Maternal characteristics**				
Age	(years)	31.7 (±4.7)	29.3 (±6.9)	**0.013** ^(1)^
Prepregnancy BMI	(kg/m^2^)	22.8 (±3.4) *n* = 97	24.8 (±5.7) *n* = 58	0.093^(2)^
Gestational weight gain	(kg)	14.8 (±3.9) *n* = 94	13.5 (±6.5) *n* = 48	**0.038** ^(2)^
Parity	(% of firstborns)	56 (55.4%)	52 (72.2%) *n* = 72	2.1 (1.1–4.0, ***p* = 0.027**)^(3)^
**At birth**				
Sex	(MALES/females) *n* (%)	52/49 (51.5/48.5%)	31/42 (42.5/57.5%)	1.4 (0.8–2.6, *p* = 0.282)^(3)^
GA	(days)	283.5 (±7.9)	277.9 (±13.4)	**0.007** ^(2)^
GA < 38 weeks	*n* (%)	2 (2%)	9 (12.3%)	7.0 (1.5–33.3, ***p* = 0.009**)^(3)^
Birth weight	(g)	3653 (±424)	3322 (±560)	**< 0.001** ^(1)^
	(SD)	−0.13 (±1.0)	−0.39 (±1.15)	**0.025** ^(2)^
Birth weight ≤ −1.25 SD	*n* (%)	12 (11.9%)	14 (19.2%)	1.8 (0.8–4.1, *p* = 0.201)^(3)^
Birth length	(cm)	50.4 (±1.9)	49.3 (±2.5)	**< 0.001** ^(2)^
	(SD)	−0.13 (±1.07)	−0.42 (±1.20)	**0.016** ^(2)^
Birth length ≤ −1.25 SD	*n* (%)	11 (10.9%)	19 (26.0%)	2.9 (1.3–6.5, ***p* = 0.014**)^(3)^
HC	(cm)	35.5 (±1.3)	34.2 (±1.9)	**< 0.001** ^(2)^
	(SD)	0.26 (±0.99)	−0.46 (±1.35)	**< 0.001** ^(1)^
HC ≤ −1.25 SD	*n* (%)	5 (5%)	17 (23.3%)	5.8 (2.0–16.7, ***p* < 0.001**)^(3)^

*Note:* Differences in weight, length, and HC of the newborns were calculated using both anthropometric measures and the SDs (*z* scores) of measures based on Finnish growth charts (Sankilampi et al. 2013). *p* values were calculated using (1) Student's *t*‐test (equal variances not assumed, two‐tailed *p* value), (2) Mann–Whitney *U* (exact two‐tailed *p* value), (3) odds ratio (OR) (95% confidence intervals [CI], Pearson chi‐squared two‐tailed *p* value). Significant *p*‐values are marked in bold.

Abbreviations: GA, gestational age; HC, head circumference.

### 2.2 Dysmorphological and Neuropediatric Assessments for 6‐Year‐Old Children

Pediatric neurologist experienced in FASD conducted detailed dysmorphological assessments, and full neuropediatric examination including testing for balance. Height, weight, HC, and palpebral fissure lengths were measured.

#### 2.2.1 Dysmorphological Assessment

Minor anomalies and notable features were recorded, and a dysmorphology score was calculated based on an adaptation of Hoyme et al. (2016). Modifications included:

1. FAS features (facial gestalt, HC, and growth) were considered separately.
2. Epicanthal folds and anteverted nares scored 1 point each due to their prevalence in Finnish children (compare Autti‐Rämö et al. (2006) and Hoyme et al. (2016)).
3. Interpupillary distance not measured due to lack of ethnicity‐specific chart.
4. Railroad track ears were replaced by any outer ear anomaly.
5. Prognathism, hirsutism, and altered palmar creases scored as “other anomalies,” 1 point each. Malocclusion due to prognathism recorded under dental malocclusion.
6. Dental malocclusion and refractive errors requiring glasses by age 6 listed as separate categories, as they are commonly recognized in FASD (Aring et al. 2021; Blanck‐Lubarsch et al. 2019; Ludwików et al. 2022; Tsang et al. 2023).

#### 2.2.2 Neuropediatric Assessment

The examination included

1. Developmental and medical history interview from parent or caregiver, including walking and talking ages;
2. Observation on behavior, attention, activity level, eye contact, interaction, cooperation, speech and understanding, movement, tremor, postures, and mental state;
3. Cranial nerve assessment (excluding olfaction);
4. Eye examination including pupil reactions, ocular movements, Hirschberg, cover test, and direct ophthalmoscopy;
5. Motor assessment including Romberg, deep tendon reflexes, Babinski, finger‐to‐nose, rapid alternating movements, muscular power and tone, range of motion of joints, gait tests, hopping, and throwing and catching a ball;
6. Static balance measured in seconds using a stopwatch as one‐foot stance with eyes open and closed, best of three trials per foot recorded;
7. Basic pediatric examination including heart and lung auscultation, otoscopy, skin and hair inspection, and neck and abdominal palpation.

### 2.3 Neuropsychological Assessments for 6‐Year‐Old Children

Neuropsychologist performed the following testing:

*WPPSI‐III (Wechsler Preschool and Primary Scale of Intelligence‐Third Edition)*: WPPSI‐III was used to assess general cognitive abilities. The following subtests were included in analysis: information, vocabulary, word reasoning, block design, matrix reasoning, picture concepts, symbol search, coding, receptive vocabulary, and picture naming. Derived scores included: full‐scale intelligence quotient (FSIQ), verbal intelligence quotient (VIQ), performance intelligence quotient (PIQ), processing speed quotient (PSQ), and general language composite (GLC) (Luiselli et al. 2013). The standard mean = 100, SD = 15. Higher scores indicate better abilities.

*WISC‐IV (Wechsler Intelligence Scale for Children‐IV)*: The following subtests were included: digit span and arithmetic. These subtests are used to calculate the working memory index (WMI) (Grizzle 2011). Standard mean = 100, SD = 15. Higher scores indicate better abilities.

For WPSSI‐III and WISC IV, the following cut‐offs were used: FSIQ < 70 for intellectual disability range, 70–84 for borderline intellectual functioning, and VIQ ≤ 70 while PIQ > 89 for performance typical for developmental language disorder. According to the IOM diagnostic criteria for FASD, the level required for cognitive impairment was defined as a score < 78 or −1.5 SD in any analyzed cognitive domain (FSIQ, VIQ, PIQ, PSQ, or WMI).

*VINELAND*: Adaptive functioning was assessed using Vineland interview for parents (Doll 1965). Scores were converted to developmental age, and a delay > 1 year was considered significant.

The following questionnaires were filled out by the child's parent(s) and preschool/kindergarten teacher:

*ADHD‐RS (ADHD Rating Scale IV)*: ADHD‐RS includes 18 items assessing symptoms of attention deficit hyperactivity disorder (ADHD) (ADHD: Current Care Guidelines 2025; DuPaul et al. 1998). Subscales are inattention (nine items) and hyperactivity/impulsivity (9 items). Maximum total score is 54. Higher scores indicate more difficulties. Scores ≥ 93rd percentile suggest likely ADHD; scores ≤ 85th percentile indicate unlikely ADHD.

*SRS (Social Responsiveness Scale)*: SRS is a 65‐item screening tool for ASD traits in the following areas: social awareness, social cognition, social communication, social motivation, and autistic mannerisms including questions about restricted interests and repetitive behavior (Constantino and Gruber 2005). Higher scores indicate more difficulties, and *T* scores ≥ 60 indicate clinically significant difficulties.

*SDQ (The Strengths and Difficulties Questionnaire)*: SDQ is a 25‐item screening tool for emotional, conduct, hyperactivity and peer problems, and prosocial behavior. Higher scores indicate greater difficulties except in the prosocial scale in which lower scores indicate greater difficulties. Each subscale has five items and produces a score between 0 and 10 (Goodman et al. 2003).

### 2.4 FASD Evaluation

FASD was assessed using modified 2016 IOM criteria for children > 3 years (Hoyme et al. 2016), which are standard in Finland, as the earlier 2005 IOM diagnostic criteria were validated for the Finnish population (Autti‐Rämö et al. 2006). Modifications include

1. Philtrum and lip assessment: Four‐Digit Code visual guide used instead of IOM version, as it offers greater specificity, though with lower sensitivity (Astley et al. 2017; Hemingway et al. 2019).
2. Growth cutoffs: *Z* scores for height and HC, and weight‐for‐height relative to the mean for age and sex used in place of the 10th percentile due to Finnish growth charts reporting those (Saari et al. 2011).

As all children in the PAE group had documented PAE, they were evaluated for

1. Growth deficiency (required in FAS): Height and HC < −1.25 SD; weight‐for‐height ratio relative to mean for age and sex < −10%.
2. Facial features (required for FAS and PFAS): Short palpebral fissures < −1.25 SD in Scandinavian chart (Strömland et al. 1999), thin upper lip, and smooth philtrum score 4 or 5 on Four‐Digit Code visual.
3. Neurobehavioral impairment (required for FAS, PFAS, and ARND): Scores < −1.5 SD (i.e., score 78) for FSIQ, VIQ, PIQ, PSQ, and WMI, as well as > 93rd percentile in ADHD‐RS score and very high range SDQ score for mood (emotional) or behavioral (conduct) regulation.
4. Malformations (required for ARBD): Clinical or medical history assessment, as no radiological examinations were performed, including congenital heart defects, radioulnar synostosis, vertebral abnormalities, kidney malformations, strabismus, ptosis, retinal vascular anomalies, optic nerve hypoplasia, and hearing loss.

### 2.5 Statistical Analysis

Analyses were performed in IBM SPSS Statistics, version 30.0 (IBM Corp. 2024). Data are presented as mean ± SD for normally distributed variables. Group differences were assessed using Student's *t*‐test or Mann–Whitney *U* test, depending on distribution and variance. Proportions were compared using odds ratios (ORs) with 95% confidence intervals (CI) and Pearson's chi‐squared test; Fisher's exact test was used when ORs could not be estimated. Spearman's rank correlation coefficient, along with two‐tailed *p* values, was used for correlation analysis. Cognitive scores were adjusted for educational level using a multivariate general linear model in IBM SPSS Statistics, with group (control vs. PAE) and the education level of the more highly educated parent as fixed factors.

## 3 Results

### 3.1 Characteristics of the Cohort

When the general characteristics of newborns and their mothers in the epiFASD cohort were compared, the control mothers were older and had higher gestational weight gain compared to the mothers of PAE children (*p* = 0.013, *p* = 0.038, respectively) (Table 1). GA was significantly shorter (*p* = 0.007), and weight, length, and HC (SDs) at birth were significantly smaller in PAE children compared to controls (*p* = 0.025, *p* = 0.016, *p* < 0.001, respectively). At birth, 8/73 (11%) of the PAE children had microcephaly (HC < −2SD), and other malformations were observed in 4/73 (5.5%) children with PAE: three had cleft lip/palate and one had polydactyly. One case of microcephaly was observed in the control group. Of the children with birth malformations, only one—a child in the PAE group who had microcephaly at birth—participated in the follow‐up at age 6.

The GA of tested controls was significantly longer compared to untested controls (*p* < 0.001) (Table S1). When anthropometrics were compared between untested PAE and control children as well as tested PAE and control children, both PAE groups had significantly smaller HCs (SD) (*p* = 0.001, *p* = 0.042, respectively). Among untested PAE children, a higher number had small HCs (HC ≤ −1.25 SD) compared to tested PAE.

### 3.2 Prenatal Exposures of the Cohort

**FIGURE 2:**
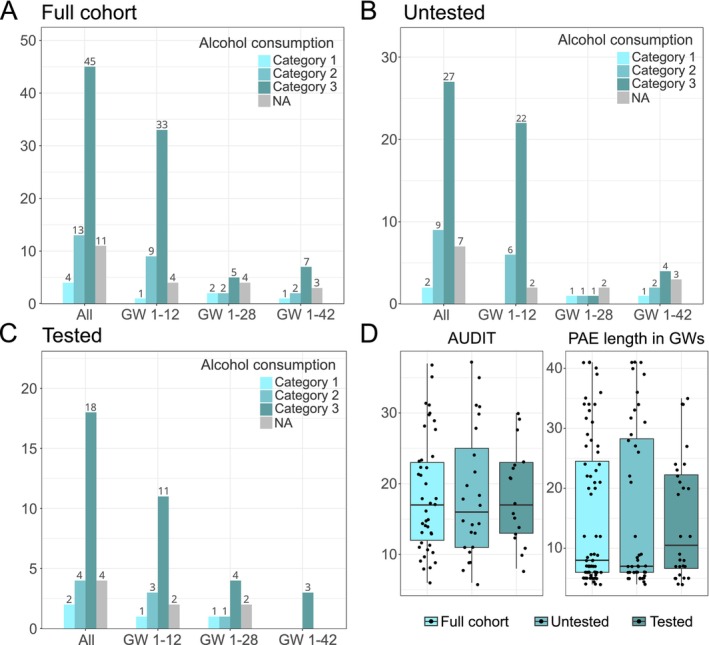
Amount and timing of maternal alcohol consumption in the whole cohort as well as in the subgroups of untested and tested PAE children. Amount and timing of maternal alcohol consumption in categories in (A) the whole cohort, (B) untested, and (C) tested subgroup. Categories for maternal alcohol consumption: AUDIT scores 1–5 suggest low‐risk consumption or < 7 alcohol units consumed per week (ad) cause low risk for morbidity and mortality for nonpregnant women (category 1), AUDIT scores 6–13 suggest hazardous or harmful alcohol consumption or 7–11 ad cause moderate risk for morbidity and mortality for nonpregnant women (category 2), and AUDIT scores 14–40 indicate the likelihood of alcohol dependence (moderate–severe alcohol use disorder) or ≥ 12 ad cause high risk for morbidity and mortality for nonpregnant women (category 3). Categories for gestational weeks (GW): GW 1–12, GW 1–28, and GW 1–42. NA, Not applicable. (D) AUDIT scores and PAE length in GWs (mean ± SD) in the full cohort (*n* = 73), untested (*n* = 45), and tested (*n* = 28) groups. No significant differences in the amount or timing of maternal alcohol consumption between groups were observed.

Alcohol consumption by mothers was substantial, particularly during the first trimester (Figure 2a–c). Both mean AUDIT score (18.5) and average maximum number of drinks per week (25.7) were in the highest alcohol use category 3 classified as high‐risk/likely alcohol dependence (Table 2). Smoking was prevalent among mothers in the PAE group: 62/73 (84.9%) of infants had at least early prenatal smoking exposure and 43/73 (58.9%) had prenatal smoking exposure throughout gestation. A significant minority of the PAE group had also co‐occurring medication or drug exposures. Controls did not have any disclosed exposures.

**TABLE 2:** Prenatal exposure information in the full cohort as well as in untested and tested children.

		Full cohort at birth (*n* = 73)	Untested at 6 years (*n* = 45)	Tested at 6 years (*n* = 28)	Tested vs. untested, *p* or OR (95% CI, *p*)
AUDIT score who had reported	*n* (%)	41 (56.2%)	24 (53.3%)	17 (60.7%)	1.4 (0.5–3.5, *p* = 0.630)^(3)^
AUDIT score	(0–40) Mean (±SD)	18.46 (±8.2)	18.50 (±9.2)	18.41 (±6.9)	0.973^(1)^
Drinks/week who had reported	*n* (%)	27 (37.0%)	18 (40%)	9 (32.1%)	0.7 (0.3–1.9, *p* = 0.620)^(3)^
Maximum drinks/week	(ad) Mean (±SD)	25.7 (±18.6)	23.6 (±16.1)	29.9 (±23.3)	0.415^(1)^
PAE length in gestation weeks	(weeks) Mean (±SD)	15.5 (±12.2) *n* = 72	15.9 (±13.4) *n* = 44	14.8 (±10.1)	0.968^(2)^
Early PAE up to GW7 at maximum	*n* (%)	33 (45.2%)	23 (51.1%)	10 (35.7%)	0.5 (0.2–1.4, *p* = 0.233)^(3)^
PAE throughout pregnancy	*n* (%)	40 (54.7%)	22 (48.9%)	18 (64.3%)	1.9 (0.7–5.0, *p* = 0.233)^(3)^
Smoking exposure (any)	*n* (%)	62 (84.9%)	37 (82.2%)	25 (89.3%)	1.8 (0.4–7.5, *p* = 0.514)^(3)^
Smoking throughout pregnancy	*n* (%)	43 (58.9%)	25 (55.6%)	18 (64.3%)	1.4 (0.6–3.8, *p* = 0.477)^(3)^
Antidepressant medication (any)	*n* (%)	12 (16.4%)	7 (15.6%)	5 (17.9%)	1.2 (0.3–4.2, *p* > 0.999)^(3)^
Benzodiazepine use (any)	*n* (%)	6 (8.2%)	3 (6.7%)	3 (10.7%)	1.7 (0.3–9.0, *p* = 0.669)^(3)^
Antipsychotic or bipolar medication (any)	*n* (%)	6 (8.2%)	5 (11.1%)	1 (3.6%)	0.3 (0.0–2.7, *p* = 0.396)^(3)^
Any self‐reported illegal drug use	*n* (%)	4 (5.5%)	3 (6.7%)	1 (3.6%)	0.5 (0.1–5.3, *p* = 0.656)^(3)^

*Note:* Data presented as mean ± SD or proportions. *p* values were calculated using (1) Student's *t*‐test (equal variances assumed, two‐tailed *p* value), (2) Mann–Whitney *U* (exact two‐tailed *p* value), (3) odds ratio (OR) (95% confidence intervals (CI), Pearson chi‐squared two‐tailed *p* value).

Abbreviations: ad, number of alcohol units consumed per week; AUDIT, alcohol use disorders identification test; GW, gestational week.

When the representativeness of the tested subgroup relative to the cohort was assessed, no significant differences in the amount or timing of maternal alcohol consumption between tested (*n* = 28) and untested (*n* = 45) pregnancies were observed (Figure 2d, Table 2). The groups did not differ significantly from each other in smoking (82.2% of untested and 89.3% of those tested were exposed to smoking) or maternal medication and drug use.

### 3.3 Characteristics of the Tested 6‐Year‐Olds

**FIGURE 3:**
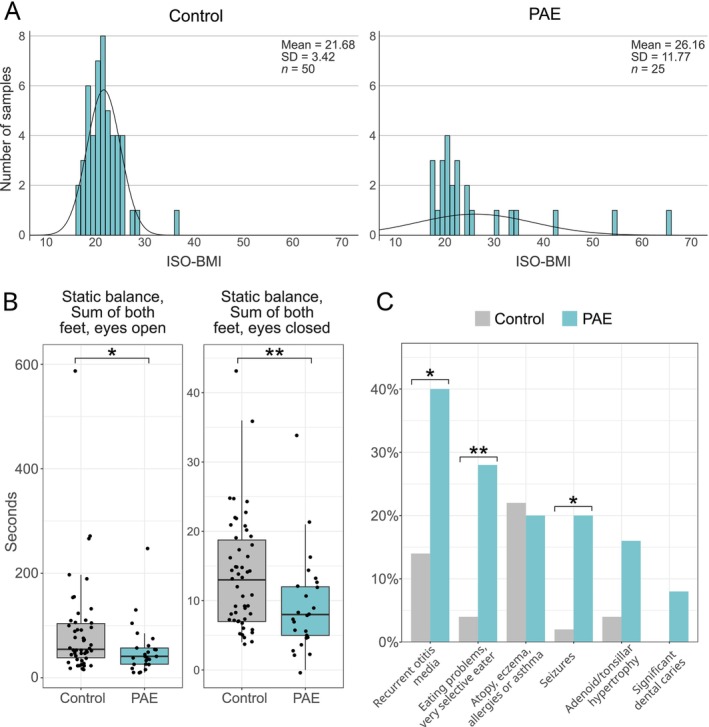
Assessment of weight status by International Standardized Body Mass Index (ISO‐BMI), static balance, and somatic conditions of 6‐year‐old PAE and control children. (A) A higher number of overweight (ISO‐BMI > 25) children was observed in the PAE group compared to controls. ISO‐BMI, childhood BMI age‐ and sex‐corrected to the adult BMI equivalent. (B) Static balance, which was measured by standing on one foot with eyes open and closed (best of three tries for each foot), was weaker in the PAE group compared to controls. (C) Increased number of somatic conditions were observed in the PAE group (eyesight problems are included in dysmorphology). *p < 0.05, **p < 0.01.

Smaller HCs (≤ −1.25 SD) were more common in the PAE group than in the control group at the age of six (OR: 6.0, 95% CI: 1.4–26.2, *p* = 0.013) (Table 3). Among control boys, only three (10.7%) were underweight and only two (7.1%) were overweight. Although PAE newborns had a trend of lower birth weight at birth, overweight (ISO‐BMI > 25) was more common in the PAE children compared to controls at the age of 6 (OR: 4.4, 95% CI: 1.2–17.2, *p* = 0.035) (Figure 3a). Due to a few significantly overweight children, the mean weight in the PAE group was higher than in controls. Only a minority of boys (3/10) in the PAE group had weight within normal limits relative to their age and height; two (20%) were underweight, and five (50%) were overweight (Figure S1).

**TABLE 3:** General characteristics of tested 6‐year‐old prenatally alcohol‐exposed (PAE) and control children.

			Control children (*n* = 52)	PAE children (*n* = 28)	*p* or OR (95% CI, *p*)
**Maternal characteristics**					
Age	(years)	Mean (±SD)	31.3 (±4.5)	29.4 (±6.2)	0.159^(1)^
Prepregnancy BMI	(kg/m^2^)	Mean (±SD)	22.8 (±3.2) *n* = 52	25.5 (±6.2) *n* = 22	0.191^(3)^
Gestational weight gain	(kg)	Mean (±SD)	14.9 (±3.7) *n* = 49	15.1 (±5.8) *n* = 19	0.899^(1)^
Parity	(% of firstborns)	*n* (%)	30 (57.7%)	20 (71.4%)	1.8 (0.7–4.9, *p* = 0.333)^(4)^
**At birth**					
Sex	(males/females)	*n* (%)	27/25 (51.9/48.1%)	11/17 (39.3/60.7%)	1.7 (0.7–4.2, *p* = 0.350)^(4)^
GA	(days)	Mean (±SD)	286.5 (±7.1)	276.4 (±14.9)	**0.002** ^(1)^
GA < 38 weeks		*n* (%)	0 (0%)	5 (17.9%)	**0.004** ^(5)^
Birth weight	(g)	Mean (±SD)	3670 (±415)	3240 (±526)	**< 0.001** ^(2)^
	(SD)	Mean (±SD)	−0.09 (±1.0)	−0.48 (±0.9)	0.088^(2)^
Birth weight ≤ −1.25 SD		*n* (%)	7 (13.5%)	4 (14.3%)	1.1 (0.3–4.0, *p* > 0.999)^(4)^
Birth length	(cm)	Mean (±SD)	50.4 (±1.6)	48.9 (±2.2)	**< 0.001** ^(3)^
	(SD)	Mean (±SD)	−0.13 (±0.90)	−0.91 (±0.96)	0.062^(2)^
Birth length ≤ −1.25 SD		*n* (%)	6 (11.5%)	8 (28.6%)	3.1 (0.9–10.0, *p* = 0.070)^(4)^
HC	(cm)	Mean (±SD)	35.6 (±1.2)	34.3 (±2.6)	**< 0.001** ^(3)^
	(SD)	Mean (±SD)	0.22 (±0.87)	−0.24 (±1.07)	**0.042** ^(2)^
HC ≤ −1.25 SD		*n* (%)	3 (5.8%)	4 (14.3%)	2.7 (0.6–13.1, *p* = 0.232)^(4)^
**At 6 years of age**			***n* = 50**	***n* = 25**	
Weight	(kg)	Mean (±SD)	22.3 (±2.9)	24.6 (±6.9)	0.559^(3)^
	(Weight‐for‐height)	Mean (±SD)	0.54 (±8.9)	7.48 (±21.17)	0.653^(3)^
	(ISO‐BMI)	Mean (±SD)	21.7 (±3.4)	26.2 (±11.8)	0.385^(3)^
Underweight	*n* (%)	*n* (%)	5 (10%)	3 (12%)	1.2 (0.3–5.6, *p* > 0.999)^(4)^
Ever been underweight	*n* (%)	*n* (%)	5 (10%)	7 (28%)	2.8 (0.8–10.5, *p* = 0.164)^(4)^
Overweight	*n* (%)	*n* (%)	4 (8%)	7 (28%)	4.4 (1.2–17.2, ***p* = 0.035**)^(4)^
Height	(cm)	Mean (±SD)	118.6 (±4.6)	119.0 (±4.9)	0.739^(2)^
	(SD)	Mean (±SD)	−0.03 (±1.42)	0.02 (±1.09)	0.831^(2)^
Height ≤ −1.25 SD	*n* (%)	*n* (%)	4 (8%)	3 (12%)	1.6 (0.3–7.6, *p* = 0.680)^(4)^
HC	(cm)	Mean (±SD)	52.6 (±1.3)	52.0 (±1.6)	0.085^(3)^
	(SD)	Mean (±SD)	0.03 (±1.0)	−0.43 (±1.3)	0.084^(2)^
HC ≤ −1.25 SD	*n* (%)	*n* (%)	3 (6%) *n* = 49	7 (28%)	6.0 (1.4–26.2, ***p* = 0.013**)^(4)^
**Family background**					
*Education of the higher‐educated parent*			***n* = 51**	***n* = 24**	
Basic education	(9 years or less)	*n* (%)	0 (0%)	3 (12.5%)	**0.029** ^(5)^
Completed secondary education	(12 years)	*n* (%)	7 (13.7%)	10 (41.7%)	4.6 (1.5–14.3, ***p* = 0.009**)^(4)^
Post‐secondary or tertiary education	(> 12 years)	*n* (%)	44 (86.3%)	11 (45.8%)	0.1 (0.0–0.4, ***p* < 0.001**)^(4)^
*Education of the other parent*			***n* = 43**	***n* = 16**	
Basic education (9 years or less)	(9 years or less)	*n* (%)	0 (%)	4 (25%)	**0.004** ^(5)^
Completed secondary education	(12 years)	*n* (%)	10 (23.3%)	5 (31.3%)	1.5 (0.4–5.4, *p* = 0.738)^(4)^
Postsecondary or tertiary education	(> 12 years)	*n* (%)	33 (76.7%)	7 (43.8%)	0.2 (0.1–0.8, ***p* = 0.027**)^(4)^
**Family history**			***n* = 52**	***n* = 25**	
Attention difficulties		*n* (%)	7 (13.5%)	8 (32%)	3.0 (1.0–9.6, *p* = 0.069)
Autism spectrum disorders		*n* (%)	1 (1.9%)	4 (16.7%) *n* = 24	10.2 (1.1–96.9, ***p* = 0.032**)
Language difficulties		*n* (%)	12 (23.1%)	6 (25.0%) *n* = 24	1.1 (0.4–3.4, *p* < 0.999)
Other learning difficulties		*n* (%)	2 (3.8%)	3 (12.5%) *n* = 24	3.6 (0.6–23.0, *p* = 0.318)
			***n* = 52**	***n* = 25**	
Bilingual/multilingual		*n* (%)	7 (13.5%)	4 (16.7%) *n* = 24	1.3 (0.4–4.9, *p* = 0.734)^(4)^
Adoption		*n* (%)	0 (0%)	4 (16%)	**0.009** ^(5)^
Foster care		*n* (%)	0 (0%)	3 (12%)	**0.031** ^(5)^

*Note:* Differences in weight, length, and HC were calculated using both anthropometric measures and the SDs (*z* scores) of measures based on Finnish growth charts (Sankilampi et al. 2013). *p* values were calculated using (1) Student's *t*‐test (equal variances not assumed, two‐tailed *p* value), (2) Student's *t*‐test (equal variances assumed, two‐tailed *p* value), (3) Mann–Whitney *U* (exact two‐tailed *p* value), (4) odds ratio (OR) (95% confidence intervals [CI], Pearson chi‐squared two‐tailed *p* value), and (5) Fisher's exact test (two‐tailed *p* value). Significant *p*‐values are marked in bold.

Abbreviations: BMI, body mass index; Ever been underweight, at 6 years or before weight for height < −10% of mean weight for height for sex; GA, gestational age; HC, head circumference; ISO‐BMI, childhood BMI age‐ and sex‐corrected to the adult BMI equivalent; overweight, ISO‐BMI > 25; underweight, ISO‐BMI < 18; Weight‐for‐height, difference in percents from mean for age and sex.

Parents of control children had higher education compared to parents of PAE children (Table 3). Eight families in the PAE group (32%) and seven families in the control group (13.5%) had a history of attention difficulties, and four families in the PAE group (16.7%) and one family in the control group (1.9%) had a history of ASD. In the PAE group, four children were adopted and three were in foster care.

### 3.4 Dysmorphological Phenotypes at Age 6

Children with PAE had significantly more dysmorphic features than controls, with both total FAS dysmorphology scores and all dysmorphology scores being notably higher in the PAE group (*p* < 0.001) (Table 4). In addition to classic FAS‐associated features, such as short palpebral fissure length, smooth philtrum, and thin vermilion, numerous minor anomalies were observed. Mild midface hypoplasia was strongly associated with PAE (OR: 40.3, 95% CI: 9.4–173.0, *p* < 0.001).

**TABLE 4:** Fetal alcohol syndrome (FAS) dysmorphology scores and other dysmorphology scores of 6‐year‐old prenatally alcohol‐exposed (PAE) and control children.

Feature	Score	Control children (*n* = 50) *n* (%)	PAE children (*n* = 25) *n* (%)	*p* or OR (95% CI, *p*)
**FAS dysmorphology**				
Head circumference < −1.25 SD	3	3 (6.1%) *n* = 49	7 (28%)	5.9 (1.4–25.7, ***p* = 0.014**)^(1)^
Weight‐for‐height < −10% of mean	1	4 (8%)	4 (16%)	2.2 (0.5–9.6, *p* = 0.429)^(1)^
Height < −1.25 SD	2	4 (8%)	3 (12%)	1.5 (0.3–7.6, *p* = 0.680)^(1)^
Short palpebral fissure length < −1.25 SD	3	2 (4.2%) *n* = 48	15 (60%)	34.5 (6.8–175.4, ***p* < 0.001**)^(1)^
Smooth philtrum grade IV or V	3	4 (8%)	13 (52%)	12.5 (3.4–45.2, ***p* < 0.001**)^(1)^
Thin vermilion grade IV or V	3	2 (4%)	11 (44%)	18.9 (3.7–95.3, ***p* < 0.001**)^(1)^
**Total FAS score** **Mean (**±**SD**)	Max 15	0.86 (±1.44)	6.08 (±3.46)	**< 0.001** ^(2)^
**Other minor anomalies**				
Hypoplastic midface	2	3 (6%)	18 (72%)	40.3 (9.4–173.0, ***p* < 0.001**)^(1)^
Long philtrum	2	3 (6%)	8 (32%)	7.4 (1.8–31.1, ***p* = 0.005**)^(1)^
Flat nasal bridge	2	4 (8%)	7 (28%)	4.5 (1.2–17.1, ***p* = 0.035**)^(1)^
Camptodactyly	2	0 (%)	1 (4%)	0.324^(3)^
Ptosis	2	2 (4%)	1 (4%)	1 (0.1–11.6, *p* > 0.999)^(1)^
5th finger clinodactyly	2	7 (14%)	7 (28%)	2.4 (0.7–7.8, *p* = 0.208)^(1)^
Strabismus	1	2 (4%)	5 (20%)	4.6 (0.8–27.0, *p* = 0.170)^(1)^
Refraction disorder, glasses at 6 years	1	3 (6%)	2 (8%)	1.4 (0.2–8.7, *p* > 0.999)^(1)^
Epicanthal folds	1	14 (28%)	14 (56%)	3.3 (1.2–8.9, ***p* = 0.024**)^(1)^
Anteverted nares	1	17 (34%)	15 (60%)	2.9 (1.1–7.9, ***p* = 0.047**)^(1)^
Limited elbow supination	1	1 (2%)	4 (16%)	9.3 (1.0–88.6, ***p* = 0.040**)^(1)^
Outer ear anomaly	1	8 (16%)	9 (36%)	3.0 (1.0–9.0, *p* = 0.078)^(1)^
Dental malocclusion	1	4 (8%)	4 (16%)	2.2 (0.5–9.6, *p* = 0.429)^(1)^
Hypoplastic fingernails	1	0 (%)	1 (4%)	0.333^(3)^
Any other minor anomaly	1 each	10 (20%)	8 (32%)	1.9 (0.6–6.0, *p* = 0.390)^(1)^
**All dysmorphology score** **Mean (**±**SD**)	Max 25	3.06 (±2.53)	12.28 (±4.51)	**< 0.001** ^(2)^

*Note:* *p* values were calculated using (1) odds ratio (OR) (95% confidence intervals [CI], Pearson chi‐squared two‐tailed *p* value), (2) Mann–Whitney *U* (exact two‐tailed *p* value), and (3) Fisher's exact test (two‐tailed *p* value). Significant *p*‐values are marked in bold.

Abbreviation: weight‐for‐height, difference in percents from mean for age and sex.

Correlations between FAS and all dysmorphology scores as well as maternal alcohol consumption (AUDIT scores), length of PAE in weeks, and anthropometrics at birth and at the age of 6 were examined (Table S2). AUDIT scores correlated significantly with all dysmorphology scores (*r* = 0.554, *p* = 0.026) and PAE duration correlated with FAS dysmorphology scores (*r* = 0.412, *p* = 0.041). Negative correlations were observed between FAS dysmorphology scores and both HC and weight (SD) at birth (*r* = −0.303, *p* = 0.008; *r* = −0.252, *p* = 0.029, respectively) and HC (SD) at 6 years (*r* = −0.351, *p* = 0.002). All dysmorphology scores correlated negatively with HC and weight (SD) at birth (*r* = −0.412, *p* = 0.041; *r* = −0.241, *p* = 0.038).

### 3.5 Neuropediatric Examination

Parental reports indicated delayed walking and phrase speech in the PAE group compared to controls. In the PAE group, 4/23 (17%), and in the control group, 3/50 (6%) of the children began walking after 15 months of age. However, this difference was not statistically significant (Table S3). More PAE children, 7/20 (35%), started speaking in phrases after 2 years of age compared to 6/49 (12%) in controls (OR: 3.9, 95% CI: 1.1–13.5, *p* = 0.042).

Static balance, both eyes open and closed, was weaker in PAE children compared to controls (*p* = 0.024, *p* = 0.006, respectively) (Figure 3b). Weak balance was more common in the PAE group, 6/25 (24%), compared to controls, 4/50 (8%), though the difference was not statistically significant (Table S3).

PAE children had increased risks of somatic conditions, including recurrent middle ear infections (OR: 4.1, 95% CI: 1.3–12.7, *p* = 0.018), very selective eating (OR: 9.3, 95% CI: 1.8–49.2, *p* = 0.005), and history of seizures (OR: 12.3, 95% CI: 1.4–111.6, *p* = 0.014) (Figure 3c, Table S4).

### 3.6 Neuropsychological Examination

#### 3.6.1 Cognitive Functioning

**FIGURE 4:**
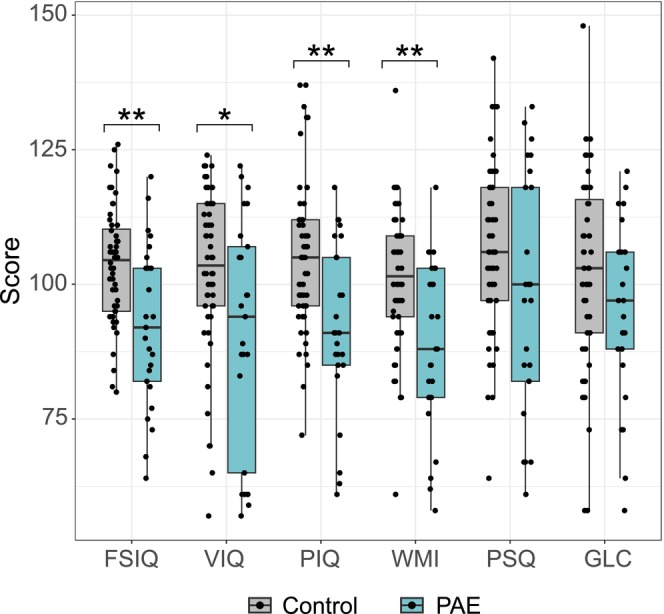
Performance of 6‐year‐old PAE and control children in cognitive tests. FSIQ, VIQ, PIQ, and WMI scores were significantly lower in PAE children compared to controls. FSIQ, Full Scale Intelligent Quotient (IQ); GLC, general language composite; PIQ, performance IQ; PSQ, processing speed quotient; VIQ, verbal IQ; WMI, Working Memory Index. *p < 0.05, **p < 0.01.

Children with PAE had lower mean cognitive test scores compared to controls (Figure 4, Table S5). FSIQ, VIQ, PIQ, and WMI scores were significantly lower in PAE children compared to controls (*p* = 0.002, *p* = 0.019, *p* = 0.001, and *p* = 0.001, respectively). In the PAE group, six children (24%) had global developmental delay corresponding to borderline intellectual functioning (FSIQ 70–84; VIQ and PIQ < 90), two children (8.3%) performed at the level of mild intellectual disability (FSIQ: 50–69), and two (8.3%) performed at the level typical for developmental language disorder (VIQ ≤ 70, PIQ > 89). No controls had global developmental delay, though four (7.7%) exhibited difficulties in language performance (VIQ ≤ 70, PIQ > 89). FASD diagnostic criteria for cognitive impairment were met in 10/25 (40%) of the PAE children, performing > 1.5 SD below the mean (< 78 IQ points) in one or more domains (FSIQ, VIQ, PIQ, WMI, or PSQ).

Due to the significantly higher educational attainment of parents of control children relative to parents of PAE children (Table 3) and previously observed associations between parental educational level and children's cognitive performance (Cermakova et al. 2023; Sirin 2005), cognitive scores were adjusted for education level using a multivariate general linear model with group (control vs. PAE) and the education level of the more highly educated parent (basic, secondary, or tertiary). FSIQ, VIQ, and GLC scores were significantly associated with parental education (*p* = 0.002, *p* < 0.001, and *p* = 0.003, respectively) when group was considered (Table S6). When parental education level was taken into account, PIQ and WMI scores remained significantly lower in PAE children compared with controls (*p* = 0.009 and *p* = 0.033, respectively) (Table S6).

Correlations between cognitive test scores and AUDIT scores, length of the PAE, anthropometrics, as well as FAS and all dysmorphology scores were examined (Table S2). Significant negative moderate correlations between FAS dysmorphology scores and FSIQ (*r* = −0.354, *p* = 0.002), VIQ (*r* = −0.241, *p* = 0.038), PIQ (*r* = −0.345, *p* = 0.003), WMI (*r* = −0.302, *p* = 0.009), and PSQ (*r* = −0.232, *p* = 0.046) were observed. Also, all dysmorphology scores correlated negatively with FSIQ (*r* = −0.334, *p* = 0.004), VIQ (*r* = −0.230, *p* = 0.049), PIQ (*r* = −0.290, *p* = 0.012), and WMI (*r* = −0.304, *p* = 0.009). Also, HC (SD) at the age of six correlated positively with PIQ (*r* = 0.261, *p* = 0.026).

#### 3.6.2 Adaptive Functioning

The difference between chronological age and socioemotional developmental age and in daily life functioning was assessed using the Vineland interview with parents (Table S7). According to Vineland interviews, the children with PAE had lower total adaptive scores and a higher proportion of lower socioemotional ages relative to chronological age (*p* < 0.001). Developmental delays > 1 year were more frequent in the PAE children (10/26, 38.5%) versus controls (1/48, 2.1%; OR: 29.4, 95% CI: 3.5–247.8, *p* < 0.001). Communication, general self‐help, and self‐help dressing scores were lower in PAE children (*p* = 0.02, *p* = 0.003, *p* = 0.003, respectively).

#### 3.6.3 Neurobehavioral and Social Functioning

**FIGURE 5:**
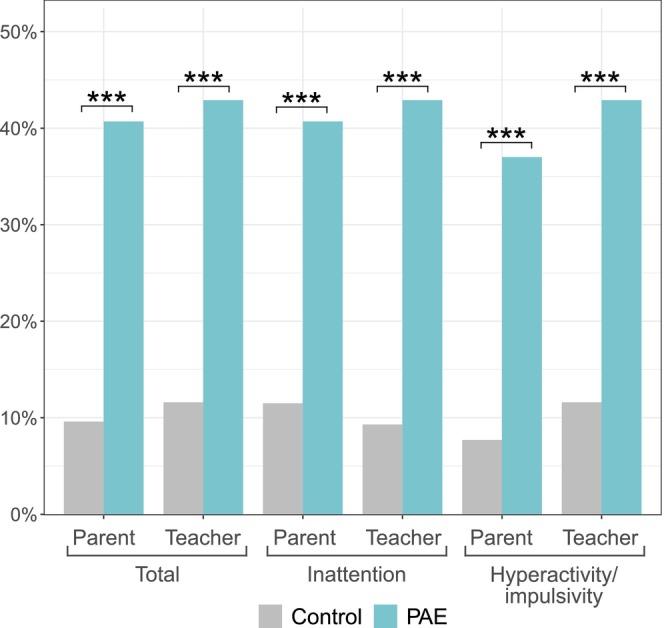
Percentages of PAE and control children having elevated (> 85th percentile) ADHD‐RS scores reported by parents and teachers in categories total, inattention, and hyperactivity/impulsivity at the age of 6. ***p value < 0.001.

ADHD symptoms, assessed using ADHD‐RS and SDQ (hyperactivity category) questionnaires completed by parents and teachers, were more prevalent in the PAE group in all categories: total, inattention, and hyperactivity/impulsivity (Figure 5, Table S8). Based on ADHD‐RS being > 93rd percentile in any category (total, inattention, or hyperactivity/impulsivity) or SDQ hyperactivity score in “very high” category, along with presenting significant attention problems and/or hyperactivity/impulsivity in neuropsychological and neuropediatric assessments, clinically significant ADHD symptoms were observed in 11/25 (44.4%) PAE children, compared to 2 (3.8%) in the control group.

FASD criteria for neurobehavioral impairment were met in 14/24 (58.3%) PAE children in at least one domain: self‐regulation, behavioral regulation, mood regulation, attention, or impulse control. Social functioning problems, not included in the FASD criteria, were more common in the PAE children according to SRS and SDQ questionnaires (Table S9). In both groups, parents noted slightly more problems than teachers.

When correlations between ADHD‐RS and AUDIT scores, length of the PAE, anthropometrics, as well as FAS and all dysmorphology scores were examined, significant positive correlation between AUDIT scores and inattention reported by parents (*r* = 0.614, *p* = 0.011) as well as between ADHD‐RS scores reported by parents and teachers and both FAS dysmorphology scores (*r* = 0.439, *p* < 0.001; *r* = 0.487, *p* < 0.001, respectively) and all dysmorphology scores (*r* = 0.328, *p* = 0.004; *r* = −0.354, *p* = 0.006, respectively) were observed (Table S2). Also, a negative moderate correlation was detected between ADHD‐RS scores reported by parents and teachers and HC (SD) at the age of 6 (*r* = −0.262, *p* = 0.025 < 0.05; *r* = −0.345, *p* = 0.007, respectively).

When correlations between total problem scores of SRS and SDQ questionnaires reported by parents and teachers as well as previously mentioned variables were calculated, a positive correlation was observed with AUDIT and SRS scores reported by parents (*r* = 0.547, *p* = 0.035) as well as both SRS and SDQ scores and FAS dysmorphology scores, all dysmorphology scores, and HC (SD) at the age of 6 (Table S2).

### 3.7 FASD Diagnoses

Based on dysmorphological and neuropsychological assessments and used questionnaires, 19/25 (76%) of the PAE children met criteria for FASD at 6 years (Table 5). Of the six children who did not meet the FASD criteria, two had facial features characteristic of FAS but no significant cognitive, behavioral, or growth impairments at this age. Two had impaired adaptive functioning, which is not included in the applied FASD criteria.

**TABLE 5:** Fetal alcohol spectrum disorder (FASD) diagnoses and other FASD‐associated features of tested 6‐year‐old prenatally alcohol‐exposed (PAE) children.

		FAS (*n* = 5)	PFAS (*n* = 6)	ARND (*n* = 7)	All FASD (*n* = 18 + ARBD)	No FASD diagnosis yet (*n* = 6)
**FASD diagnostic criteria**						
FAS dysmorphic score	Mean (±SD)	10.4 (±1.9)	7.5 (±1.6)	4.0 (±3.1)	6.7 (±3.5)	4 (±2.5)
Growth impairment	*n* (%)	5 (100%)	0 (0%)	3 (42.9%)	9 (47.4%)	0 (0%)
HC < −1.2 SD	*n* (%)	4 (80%)	2 (33.3%)	1 (14.2%)	7 (36.8%)	0 (0%)
Cognitive impairment*	*n* (%)	2 (50%) *n* = 4	3 (50%)	5 (71.4%)	10 (52.6%)	0 (0%)
FSIQ	Mean (±SD)	87.0 (±9.1)	87.3 (±17.5)	86.4 (±11.9)	88.5 (±14.2)	106.5 (±7.4)
Behavioral impairment	*n* (%)	5 (100%)	4 (66.7%)	5 (71.4%)	14 (73.7%)	0 (0%)
ADHD	*n* (%)	4 (80%)	3 (50%)	4 (57.1%)	11 (57.9%)	0 (0%)
ADHD‐RS total score (parent)	Mean (±SD)	33.8 (±10.1)	20.3 (±6.1)	24.3 (±12.0)	23.8 (±11.2)	10.3 (±5.8)
**Other FASD‐associated features**						
History of any seizures, EEG performed	*n* (%)	2 (40%)	1 (16.7%)	0 (0%)	5 (26.3%)	0 (0%)
Total dysmorphic score	Mean (±SD)	17.8 (±3.8)	13.3 (±2.9)	9.7 (±4.0)	13.1 (±4.6)	9.7 (±3.1)
Social impairment**	*n* (%)	2 (40%)	4 (66.7%)	5 (71.4%)	15 (79.0%)	0 (0%)
Adaptive impairment***	*n* (%)	4 (80%)	1 (16.7%)	2 (28.6%)	7 (36.8%)	2 (33.3%)
Coordination disorder (F82)	*n* (%)	1 (20%)	2 (33.3%)	1 (14.2%)	4 (21.1%)	2 (33.3%)
Eyesight abnormality	*n* (%)	2 (40%)	1 (16.7%)	2 (28.6%)	6 (31.6%)	0 (0%)
AUDIT score	Mean (±SD)	22.5 (±8.1)	21.7 (±1.5)	16.3 (±5.9)	20.1 (±6.8) *n* = 13	11.7 (±3.2) *n* = 3
PAE length weeks	Mean (±SD)	24.2 (±10.8)	16.2 (±12.5)	10.1 (±7.8)	15.4 (±11.4)	12.2 (±6.4)
Early only exposure	*n* (%)	0 (0%)	3 (50%)	4 (57.1%)	8 (42.1%)	2 (33.3%)

*Note:* All FASD category consists of fetal alcohol syndrome (FAS), partial FAS (PFAS), alcohol‐related neurodevelopmental disorder (ARND), and alcohol‐related birth defect (ARBD) diagnoses. *Cognitive impairment = in at least one domain < −1.5 SD or < 78 points, one child did not participate in neuropsychological evaluation. **Total Social Responsiveness Scale (SRS) *T*‐score > 59, parent's strengths and difficulties questionnaire (SDQ) peer difficulties > 4, teacher's SDQ peer difficulties > 5, parent's SDQ prosocial < 6, or teacher's SDQ prosocial < 4. ***According to the Vineland interview, the individual's socioemotional developmental age was more than 1 year below the chronological age.

Abbreviations: ADHD, attention deficit hyperactivity disorder; ADHD‐RS, ADHD rating scale; AUDIT, alcohol use disorders identification test; EEG, electroencephalogram; FSIQ, Full Scale Intelligent Quotient; HC, head circumference.

To examine the timing of maternal alcohol consumption in relation to FASD diagnoses, early PAE was defined as exposure up to GW 7. Of the early PAE subgroup, 8/11 (72.7%) were diagnosed with FASD. Heavy alcohol consumption was more prevalent in the early PAE group than in the longer PAE (Figure S2). None of the children in the early PAE subgroup were diagnosed with FAS, three had PFAS, four met the criteria of ARND, and one had ARBD. Considering GA and sex when calculating *Z* scores (SD values), neither the children in the early PAE subgroup (*n* = 11) nor the children in the longer PAE subgroup (*n* = 17) differed significantly in anthropometric measurements from the control children at birth (SDs) (Table S10a). However, in the longer PAE group, more 6‐year‐olds had been underweight, overweight, or had smaller HCs compared to the controls (≤ −1.25 SD) (OR: 4.5, 95% CI: 1.1–18.6, *p* = 0.043; OR: 5.8, 95% CI: 1.3–25.3, *p* = 0.025; OR: 10.2, 95% CI: 2.2–48.6, *p* = 0.004), respectively.

Upon further analysis of the timing of PAE and associated dysmorphology, similar rates of dysmorphic features were observed in both the early and longer exposure groups (Table S10b). In terms of cognitive performance, half of the PAE children (4/8, 50%) with borderline or lower FSIQ scores (FSIQ < 85) belonged to the early PAE group (Table S10c).

## 4 Discussion

This is a prospective longitudinal birth cohort study in which neuropsychological, neuropediatric, and dysmorphological features were examined in exceptional detail in 6‐year‐old children prenatally exposed to high levels of alcohol. Although previous prospective cohort studies have consistently shown that PAE is associated with a broad spectrum of neurodevelopmental impairments (Chu et al. 2022) as well as dysmorphological features (Jacobson et al. 2021; Muggli et al. 2025), comprehensive, simultaneous assessments across developmental stages are necessary to clarify the age‐specific FASD phenotype and improve the diagnosis of alcohol‐related developmental disorders. Given the developmental and educational importance of this stage, assessments at this age remain very limited under current diagnostic guidelines. Furthermore, we have, for the first time, comprehensively examined how early alcohol exposure—limited to the first 7 weeks of pregnancy—affects the child's later phenotype. Delayed recognition of pregnancy until approximately GW 7 is relatively common, which may result in unintentional exposure (Branum and Ahrens 2017; Floyd et al. 1999). Considering that these first GWs include implantation, gastrulation, and the beginning of organogenesis, they are highly important stages of development.

As expected, children with PAE showed a higher prevalence of dysmorphic features, neurobehavioral impairments, and somatic problems compared with controls. Dysmorphological evaluation demonstrated a broad range of minor anomalies beyond the classical features of FAS. The negative correlation between dysmorphology scores and both HC and weight (SD) at birth and at the age of 6 suggests that the severity of dysmorphology reflects impaired fetal development resulting from PAE. Importantly, dysmorphic features and neurobehavioral impairments were observed not only following prolonged exposure but also after substantial PAE confined to very early gestation, underscoring the sensitivity of early embryonic development to alcohol.

Unexpectedly, PAE was associated not only with underweight but also with an increased risk of being overweight among 6‐year‐olds, particularly among boys. The increased overweight risk challenges the traditional emphasis on growth restriction. However, previous studies have shown that adolescent girls with FASD have an elevated risk of overweight (Fuglestad et al. 2014), and that abnormal eating behaviors and impaired satiety mechanisms are common in children with FASD (Amos‐Kroohs et al. 2016), which may contribute to the development of overweight. However, given the limited sample size, these findings should be validated in future studies.

Children with PAE showed lower mean performance on cognitive tests compared with the control group, and several met the criteria for ARND, presenting only the neurobehavioral phenotype of FASD. ADHD‐related symptoms represented the most common form of neurobehavioral impairment. This is consistent with previous studies indicating that ADHD is the most prevalent symptom associated with FASD (Burd 2016; Clark et al. 2024). The earliest age at which ADHD can be diagnosed with reasonable reliability is approximately 6 years, and most individuals with ADHD related to PAE receive their diagnosis at a later stage (Clark et al. 2024). These findings highlight the need for continued clinical monitoring of PAE children and the importance of identifying PAE‐related difficulties that may not yet meet the formal FASD criteria at that age.

When examining the timing of maternal self‐reported alcohol consumption relative to FASD diagnoses, none of the 6‐year‐olds exposed to alcohol up to GW 7 showed growth restriction, precluding FAS diagnosis; they were instead diagnosed with PFAS or ARND. However, children with early‐only PAE showed similar rates of dysmorphic features and neurocognitive impairment compared to the longer exposure group. Four out of eight children in the PAE group with low FSIQ scores belonged to the early exposure subgroup. These findings represent rare human evidence that substantial alcohol exposure confined to very early pregnancy can be sufficient to result in diagnosable FASD with clinically significant cognitive impairment. This emphasizes the vulnerability of early pregnancy for alcohol exposure, particularly the sensitivity of the developing nervous system, and is consistent with earlier studies with human cell and animal models (Legault et al. 2021; Wallén et al. 2025). This is also consistent with guidelines recommending the use of contraception when consuming alcohol and advising individuals to stop drinking alcohol when planning a pregnancy (Carson et al. 2010). Recently, it has been shown that as a membrane‐permeable molecule, ethanol can influence embryonic and placental development even prior to implantation (Legault et al. 2021).

Alcohol exposure levels were substantial among study cohort, typically at the range of high risk for alcohol dependence and health problems. Considering the heavy exposure in this prospectively studied cohort, the high prevalence of FASD among the assessed children was expected. In a previous prospective cohort study, up to 80% of heavily exposed children exhibited at least one adverse outcome by school age, primarily reflecting central nervous system dysfunction (Kuehn et al. 2012). The mean maternal AUDIT score in the early‐only PAE group was also high, indicating that exposure intensity, even over a short period, is clinically relevant. Nevertheless, children who have not yet shown clear difficulties may develop more pronounced learning and behavioral problems as academic and social demands increase. Notably, none of the children who participated in testing had previously received an FASD diagnosis despite exhibiting clear symptoms. This highlights both the advantages of a prospective study design and the extent of underdiagnosis in healthcare. Assessing children at 6 years offers practical advantages for cooperation and planning early school‐based support. Many in‐school FASD prevalence studies have assessed children in the first grade (approximately ages 6–7).

In addition to confirming the association between PAE and cognitive impairment and ADHD symptoms included in the IOM 2016 diagnostic criteria (Hoyme et al. 2016), we also identified several other PAE‐related features. These included lower performance on static balance tasks, consistent with earlier findings (Lucas et al. 2014). Moreover, PAE increased risks for many dysmorphologies as well as ear infections, very selective eating, and problems in eyesight, self‐help skills and social functioning, not included in the used IOM 2016 diagnostic criteria but recognized as comorbidities (Attell et al. 2025; Hoyme et al. 2016; Popova et al. 2016). Even though adaptive functioning is not included in the used diagnostic criteria for FASD, it has been shown to be more affected by PAE than IQ scores in neuropsychological testing (Fagerlund et al. 2012; Streissguth et al. 2004). However, both adaptive and social functioning problems have been included in the DSM‐5 ND‐PAE (Neurobehavioral Disorder associated with PAE), which may capture ARND‐like phenotypes more comprehensively than the IOM 2016 criteria.

Several different diagnostic guidelines for FASD are in use (Hemingway et al. 2019; Myers et al. 2025), and current diagnostic guidelines predominantly emphasize the characteristic facial features of FAS due to their relative specificity. However, experimental studies have demonstrated that even a single ethanol exposure during mouse gastrulation—which corresponds to the fifth week of human gestation—can induce craniofacial abnormalities reminiscent of those seen in humans with FAS (Lipinski et al. 2012). Considering this as well as the high prevalence of ARND, concentrating only on certain dysmorphological traits may fail to capture important outcomes of exposure. Since PAE before and after gastrulation leads to different types of facial anomalies as well as changes in neurodevelopment and brain morphology (Lipinski et al. 2012; Muggli et al. 2025), the typical FAS facial features do not reflect the full extent of dysmorphology or other developmental disorders caused by exposure. As a result, the choice of diagnostic criteria can affect both the proportion and the phenotype of PAE children identified with FASD (Hemingway et al. 2019; Myers et al. 2025), highlighting the importance of comprehensively reporting the phenotypic characteristics observed in the PAE population relative to unexposed controls.

This study has several limitations. The relatively small sample size limits statistical power and prevented sex‐specific analyses. Although the PAE group contained more females, the direction of this imbalance is unlikely to explain the observed findings. Self‐reported alcohol use may underestimate true exposure, as many mothers underreport their consumption due to stigma or fear of social repercussions (Gomez‐Roig et al. 2018; Lange et al. 2014) and some unreported alcohol exposure is possible also in controls. However, the consistency between reported exposure, AUDIT scores, dysmorphology, and neurobehavioral outcomes supports data validity. It is important to note that the AUDIT was developed to assess the risk of alcohol use in nonpregnant individuals, not the risk to the fetus, and even alcohol consumption classified as Category 1 (“low risk for non‐pregnant women”) may harm the developing fetus. In this study, alcohol exposure levels were high even by AUDIT standards, particularly in the early‐only PAE group, and the results cannot be generalized to all cases of inadvertent early exposure before pregnancy is recognized. The findings also reflect combined exposure to alcohol and smoking. Because nearly all participants in the PAE group were also exposed to tobacco, it was not possible in this study to separate the effects of PAE from those of tobacco exposure. Furthermore, selection bias is possible, as families of control children having developmental concerns may have been more likely to participate, whereas parents with ongoing substance use were harder to reach. Additionally, parents of many children who already had support in place chose not to participate or were ineligible, as children had recent neuropsychological tests conducted. Unfortunately, due to variations in age and the methods used, we were unable to incorporate the data from children tested elsewhere into our analyses. When the representativeness of the tested children relative to the cohort was assessed, only significant differences were higher number of small HCs and other malformations at birth among untested PAE children. It is unlikely that untested children in PAE cohort have been exposed to lower amounts of alcohol or consist of less affected children. Based on this, we can conclude that the group of children tested is representative of the epiFASD cohort and the majority of PAE children in the whole cohort would meet diagnostic criteria for FASD.

Six years of age may not be optimal for detecting all PAE‐related challenges. The tests were conducted before school entry—age seven in Finland—and many learning difficulties as well as attention or executive function deficits often do not become apparent until formal schooling begins and demands for independent functioning increase. At the age of 6, the family environment plays a significant role in a child's development. Adoption and twin studies have shown that during early childhood, environmental factors have significantly more impact on IQ than in later life, when congenital factors outweigh them (Bouchard Jr 2013; Haworth et al. 2010). It is also important to acknowledge the influence of genetic background, especially due to reported attention difficulties and ASD among family members. Due to the significantly higher educational level of control parents, we adjusted cognitive scores by the education level of the more highly educated parent. After adjustment, the PIQ and WMI scores of PAE children remained significantly lower than those of controls, but statistically significant differences between FSIQ and VIQ did not persist. However, due to the limited sample size, these aspects could not be examined adequately in the current study, but they represent important issues to be addressed in future research.

In conclusion, in this prospective longitudinal birth cohort study, we not only confirmed robust associations between PAE and dysmorphic features, cognitive impairment, and ADHD symptoms, but also identified additional somatic, social, and adaptive difficulties not captured by the current IOM 2016 FASD criteria. These findings emphasize the complex and multidimensional nature of FASD and the need to refine diagnostic frameworks to fully capture its broader phenotype. Importantly, the observation that alcohol exposure limited to the first 7 weeks of gestation can lead to measurable dysmorphological and neurocognitive effects underscores the vulnerability of early embryonic development to alcohol and reinforces public health messages advocating complete abstinence when pregnancy is possible.

## Author Contributions

M.J.: conceptualization, data curation, investigation, formal analysis, methodology, writing – original draft, writing – review and editing. E.W.: conceptualization, data curation, investigation, formal analysis, visualization, writing – review and editing. E.S.: conceptualization, data curation, investigation, methodology, writing – review and editing. K.R.: data curation, visualization, writing – review and editing. H.K.: conceptualization, project administration, resources, writing – review and editing. N.K.‐A.: conceptualization, data curation, investigation, methodology, funding acquisition, project administration, resources, supervision, writing – review and editing.

## Funding

This project was supported by The Foundation for Pediatric Research (N. K.‐A., E.W., K.R.), The Finnish Foundation for Alcohol Studies (N.K.‐A.), Research Council of Finland (grant no.: 332212, N.K.‐A.), Jane and Aatos Erkko Foundation (grant no. 230032, N.K.‐A.), Instrumentarium Science Foundation (E.W.), Märta Klaus and Peter Klaus Foundation (N.K.‐A., K.R.), and Liv och Hälsa Foundation (N.K.‐A.). Open access was funded by Helsinki University Library.

## Conflicts of Interest

The authors declare no conflicts of interest.

## Supporting information


**Figure S1:** Assessment of 6‐year‐olds weight status by International Standardized BMI.
**Figure S2:** Timing and amount of maternal alcohol consumption in categories.

**Table S1:** General characteristics of the prenatally alcohol‐exposed (PAE) and control untested and tested children and their mothers. Differences in weight, length, and HC of the newborns were calculated using both anthropometric measures and the SDs (*Z* scores) of measures based on Finnish growth charts (Sankilampi et al. 2013). Data presented as mean ± SD or proportions. *p* values were calculated using (1) Student's *t*‐test (equal variances not assumed, two‐tailed *p* value), (2) Student's *t*‐test (equal variances assumed, two‐tailed *p* value), (3) Mann–Whitney *U* (exact two‐tailed *p* value), (4) odds ratio (OR) (95% confidence intervals (CI), Pearson chi‐squared two‐tailed *p* value), and (5) Fisher's exact test (two‐tailed *p*‐value). BMI, body mass index; GA, gestational age; HC, head circumference.
**Table S2:** Correlations between prenatal alcohol exposure (PAE) information as well as maternal, newborn and 6‐year‐old characteristics by Spearman's rank correlation. Correlations between AUDIT score, PAE length in gestational weeks (GWs), total FAS score, all dysmorphology score, HCs (SDs) at birth and at the age of 6, maternal characteristics, newborn, and 6‐year‐old phenotype as well as neuropsychological examination results by Spearman's rank correlation. Significant correlation (*p* < 0.05) are marked in green. *p < 0.05, **p < 0.01, ***p < 0.001. ADHD‐RS, ADHD Rating Scale IV; AUDIT, alcohol use disorders identification test; BMI, body mass index; FAS, fetal alcohol syndrome; FSIQ, Full Scale Intelligent Quotient (IQ); GA, gestational age; GLC, general language composite; HC, head circumference; ISO‐BMI, childhood body mass index (BMI) age‐ and sex‐corrected to the adult BMI equivalent; PIQ, performance IQ; PSQ, processing speed quotient; SDQ, strengths and difficulties; SRS, Social Responsiveness Scale; VIQ, verbal IQ; WMI, Working Memory Index.
**Table S3:** Static balance as well as walking and talking age of 6‐year‐old prenatally alcohol‐exposed (PAE) and control children. Examination results of the static balance, which was measured by standing on one foot with eyes open and closed (best of three tries for each foot, presented as sum of both feet) as well as walking and talking ages. Data presented as mean (±SD) and proportions. *p* values were calculated using (1) Mann–Whitney *U* (exact two‐tailed *p* value) or (2) odds ratio (OR) (95% confidence intervals (CI), Pearson chi‐squared two‐tailed *p* value).
**Table S4:** Somatic health of 6‐year‐old prenatally alcohol‐exposed (PAE) and control children. Proportions of somatic health conditions in 6‐year‐old PAE and control children. Eyesight problems are included in dysmorphology. *p* values were calculated using (1) odds ratio (OR) (95% confidence intervals [CI], Pearson chi‐squared two‐tailed *p* value), and (2) Fisher's exact test (two‐tailed *p* value). EEG, electroencephalogram.
**Table S5:**. Performance of 6‐year‐old prenatally alcohol‐exposed (PAE) and control children in cognitive tests. Performance scores and proportions < 80 (threshold for significant developmental delay in Finland) and < 85 (cutoff for borderline intellectual functioning [BIF]) of 6‐year‐old PAE and control children in cognitive tests. Performance scores presented as mean (range, ±SD). *p* values were calculated using (1) Student's *t*‐test (equal variances not assumed, two‐tailed *p* value), (2) Student's *t*‐test (equal variances assumed, two‐tailed *p* value), (3) odds ratio (OR) (95% confidence intervals [CI], Pearson chi‐squared two‐tailed *p* value), and (4) Fisher's exact test (two‐tailed *p* value). FSIQ, Full Scale Intelligent Quotient; GLC, general language composite; PIQ, performance IQ; PSQ, processing speed quotient; VIQ, verbal IQ; WMI, Working Memory Index.
**Table S6:** Adjustment of cognitive scores for educational level using a multivariate general linear model. Cognitive scores were adjusted for educational level using a multivariate general linear model in IBM SPSS Statistics, with group (control vs. PAE) and the education level of the more highly educated parent as fixed factors. In models with education level as predictor, the effects of group are adjusted, and in models with group as predictor, the effects of education level are adjusted. FSIQ, Full Scale Intelligent Quotient (IQ); GLC, general language composite; partial η^2^, partial eta‐squared; PIQ, performance IQ; PSQ, processing speed quotient; VIQ, verbal IQ; WMI, Working Memory Index.
**Table S7:** Adaptive functioning of 6‐year‐old prenatally alcohol‐exposed (PAE) and control children. Adaptive daily life functioning according to Vineland interview of PAE and control children at the age of 6. Differences between socioemotional age and chronological age as well as different scores presented as mean (±SD). *p* values were calculated using (1) Mann–Whitney *U* (exact two‐tailed *p* value) or (2) odds ratio (OR) (95% confidence intervals [CI], Pearson chi‐squared two‐tailed *p* value).
**Table S8:** Attention deficit hyperactivity disorder (ADHD) symptoms of 6‐year‐old prenatally alcohol‐exposed (PAE) and control children. ADHD symptoms according to the results of ADHD rating scale (ADHD‐RS) and Strengths and Difficulties Questionnaire (SDQ) hyperactivity questionnaires of the PAE and control children at the age of 6. ADHD‐RS total, inattention, and hyperactivity/impulsivity scores reported by parents and teachers presented as mean (range, ±SD). Those children who fulfilled ADHD criteria are presented in proportions based on the sex‐specific percentile thresholds. **93 percentile: girls 24 (points), boys 30; 85 percentile: girls 18, boys 22. **93 percentile: girls 12, boys 15; 85 percentile: girls 8, boys 11. ***93 percentile: girls 13, boys 17; 85 percentile: girls 9, boys 12. *p* values were calculated using (1) Mann–Whitney *U* (exact two‐tailed *p* value), (2) odds ratio (OR) (95% confidence intervals [CI], Pearson chi‐squared two‐tailed *p* value), and (3) Fisher's exact test (two‐tailed *p* value).
**Table S9:** Social functioning of 6‐year‐old prenatally alcohol‐exposed (PAE) and control children. Social functioning according to Social Responsiveness Scale (SRS) and Strengths and Difficulties (SDQ) questionnaires of the PAE and control children at the age of 6. SRS total as well as SDQ prosocial and peer difficulties scores reported by parents are presented as mean (range, ±SD). The SRS raw scores were converted to sex‐normed *T* scores based on normative data. Social impairment was defined as meeting any of the cutoff criteria (total SRS *T* score > 59, parent's SDQ prosocial < 6, or teacher's SDQ prosocial < 4, parent's SDQ peer difficulties > 4, teacher's SDQ peer difficulties > 5). *p* values were calculated using (1) Student's *t*‐test (equal variances not assumed, two‐tailed *p* value), (2) Mann–Whitney *U* (exact two‐tailed *p* value), (3) odds ratio (OR) (95% confidence intervals (CI), Pearson chi‐squared two‐tailed *p* value), and (4) Fisher's exact test (two‐tailed *p* value).
**Table S10:** Early (children exposed up to gestational week (GW) 7) and longer prenatal alcohol exposure (PAE) (exposed longer than seven first GWs) comparisons. (a) General characteristics of the PAE and control children at birth and at 6 years of age as well as PAE and family background information categorized early (children exposed up to GW 7) and longer PAE (exposed longer than seven first GWs) groups. Differences in weight, length, and HC were calculated using both anthropometric measures and the SDs (*Z* scores) of measures based on Finnish growth charts (Saari et al. 2011; Sankilampi et al. 2013). Data presented as mean ± SD or proportions. *p* values were calculated using (1) Student's *t*‐test (equal variances not assumed, two‐tailed *p*‐value), (2) Student's *t*‐test (equal variances assumed, two‐tailed *p* value), (3) Mann–Whitney U (exact two‐tailed *p* value), (4) odds ratio (OR) (95% confidence intervals [CI], Pearson chi‐squared two‐tailed *p*‐value), and (5) Fisher's exact test (two‐tailed *p* value). (b) FAS dysmorphology scores and other dysmorphology scores of 6‐year‐old PAE and control children categorized early and longer PAE groups. *p* values were calculated using (1) OR (95% CI, Pearson chi‐squared two‐tailed *p* value), (2) Mann–Whitney U (exact two‐tailed *p*‐value), (3) Student's *t*‐test (equal variances assumed, two‐tailed *p* value), and (4) Fisher's exact test (two‐tailed *p* value). (c) Performance of 6‐year‐old PAE (categorized early and longer PAE groups) and control children in cognitive tests. Performance scores and proportions < 80 (threshold for significant developmental delay in Finland) and < 85 (cutoff for boderline intellectual functioning [BIF]) of 6‐year‐old control and PAE children in cognitive tests. *p* values were not reported due to the small sample size. ad, number of alcohol units consumed per week; AUDIT, alcohol use disorders identification test; BMI, body mass index; FSIQ, Full Scale Intelligent Quotient (IQ); GA, gestational age; GLC, General Language Composite; HC, head circumference; ISO‐BMI, childhood BMI age‐ and sex‐corrected to the adult BMI equivalent; overweight, ISO‐BMI > 25; PIQ, performance IQ; PSQ, processing speed quotient; underweight, ISO‐BMI < 18; weight‐for‐height, difference in percents from mean for age and sex; VIQ, verbal IQ; WMI, Working Memory Index.

## Data Availability

The data that support the findings of this study are available from the corresponding author upon reasonable request.
