# Supplementary material for: Dysmorphological and Neuropsychological Phenotypes of Prenatally Alcohol‐Exposed 6‐Year‐Old Children: A Prospective Longitudinal Birth Cohort Study
Source: Alcohol Clin Exp Res (Hoboken). 2026 May 13;50:e70320. doi: 10.1111/acer.70320 (PMC13169235; doi:10.1111/acer.70320)
Supplement: Supplementary file 1 — Figure S1: Assessment of 6‐year‐olds weight status by International Standardized BMI. Figure S2: Timing and amount of maternal alcohol consumption in categories. [file ACER-50-0-s002.pdf]

SUPPLEMENTARY FIGURES

Dysmorphological and neuropsychological phenotypes of prenatally alcohol-exposed six-year-old children

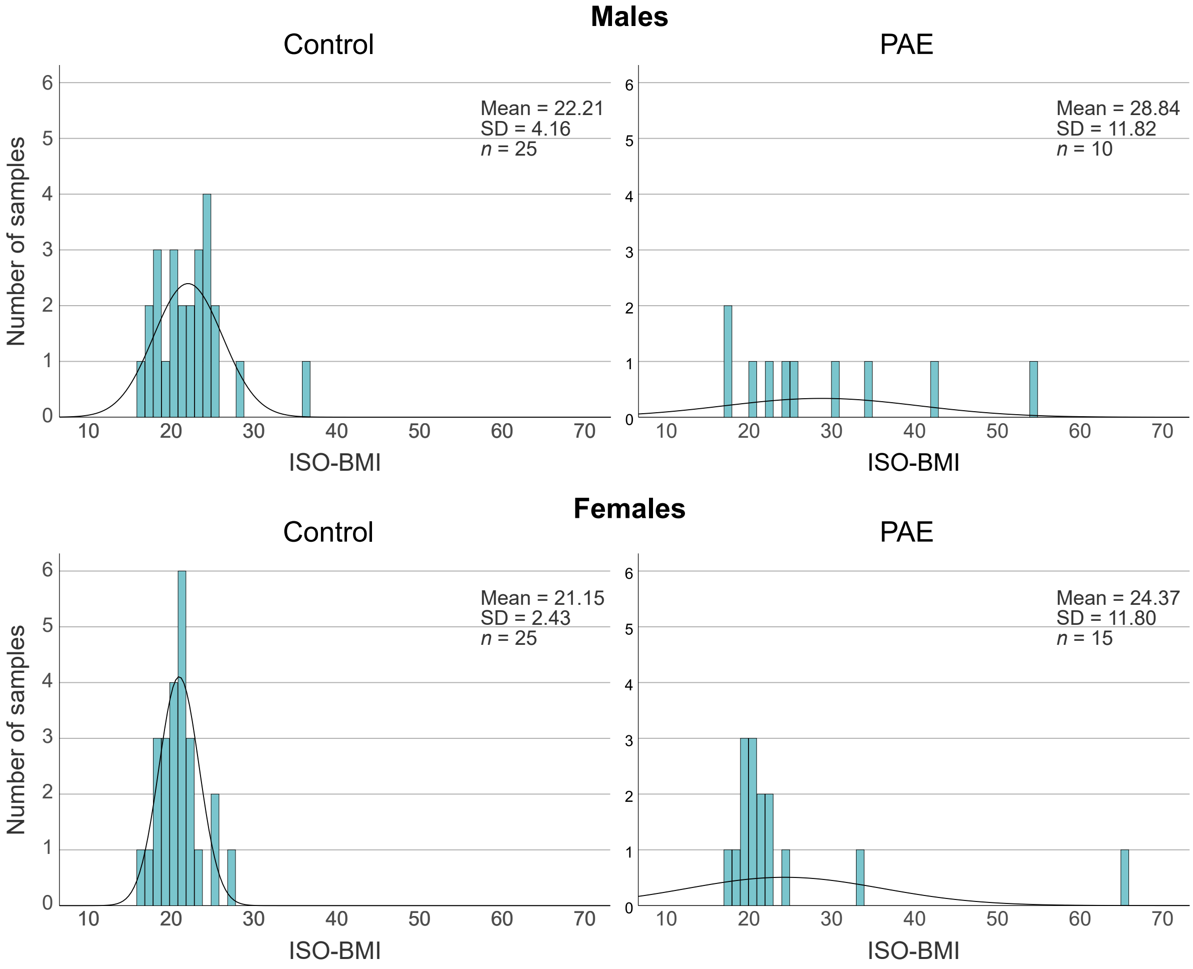

Supplementary Figure 1. Assessment of six-year-olds weight status by International Standardized Body Mass Index (ISO-BMI) in males and females. A higher number of overweight (ISO-BMI > 25) children, especially males, was observed in the PAE group compared to controls.

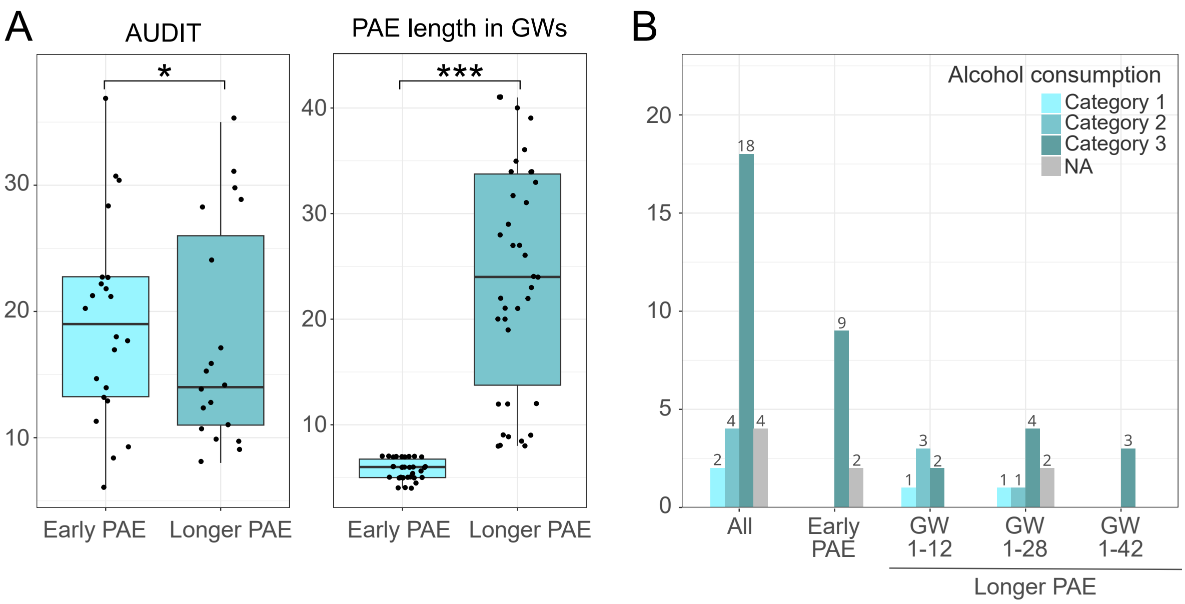

Supplementary Figure 2. Timing and amount of maternal alcohol consumption in categories. **A)** AUDIT scores and PAE length in GWs (mean ±SD) in the early and longer PAE subgroups. **p*-value <0.05. ****p*-value <0.001. **B)** Visual presentation of the timing and amount of alcohol consumption of mothers in the tested PAE group (n = 80) during whole gestation, gestational weeks (GW) 1-8 (early PAE group), GW 1–12, GW 1–28, and GW 1–42. Categories for maternal alcohol consumption: AUDIT scores 1–5 suggest low-risk consumption or < 7 alcohol units consumed per week (ad) cause low risk for morbidity and mortality for non-pregnant women (category 1), AUDIT scores 6–13 suggest hazardous or harmful alcohol consumption or 7–11 ad cause moderate risk for morbidity and mortality for non-pregnant women (category 2), and AUDIT scores 14–40 indicate the likelihood of alcohol dependence (moderate-severe alcohol use disorder) or ≥ 12 ad cause high risk for morbidity and mortality for non-pregnant women (category 3).
